# Preventing the Worst, Recovering with Resilience

**DOI:** 10.31662/jmaj.2022-0151

**Published:** 2022-09-12

**Authors:** Soichiro Saeki

**Affiliations:** 1 Department of Emergency Medicine and Critical Care, Center Hospital of the National Center for Global Health and Medicine, Tokyo, Japan

**Keywords:** Firearms, Gun Violence, Public Health, Japan

## Abstract

On July 8, 2022, Shinzo Abe, the former Prime Minister of Japan, passed away. This tragedy may turn out to have a deep impact on public health throughout the world, not limited to Japan.

According to the most recent Japanese government reports, Mr. Abe was murdered with a firearm. This could spark a new debate in the current global debate over gun control, with far-reaching consequences for public health around the world. Furthermore, extensive media coverage may harm the mental health of Japanese civilians, and such issues should be addressed in a fair manner.

The Japanese have been strong against previous disasters and tragedies. It is hoped that Japan can build back a safe and resilient society for all.

On July 8, 2022, Shinzo Abe, the former Prime Minister of Japan, passed away. He was shot from behind while giving a speech to the public for the upcoming national election ^(1)^. As a Japanese national, the author is heartbroken by this devastating news. However, the author would like to emphasize the importance of several public health issues that may arise from this case.

Firstly, we must reaffirm the dangers of firearms and weapons. Firearm-related deaths are extremely rare in Japan ^(2)^. Bearing firearms is strictly prohibited in Japan ^(1)^, and Japan has been known to be a fairly safe country. Japan remains a safe country in terms of firearm-related homicide, and this case should not be viewed as a flaw in the public health strategy of banning the use of firearms. Discussions on the ban of firearms are currently being widely discussed, especially in the United States ^(3)^, and we fear that this case may ignite opinions that try to persuade the argument that prohibiting firearms does not save lives. The safety of Japan under the ban of firearms ^(2)^ does not change by this case alone.

Secondly, healthcare professionals should take this opportunity to prepare for possible cases of injuries from firearms. Medical professionals should seize on this event to reinforce their response to gun trauma. It has been noted that widespread media coverage could inspire other crimes, and as this case has been extensively covered by the international media, such instances could spread to other countries as well. We must be better prepared than ever, particularly in Japan, where cases of injuries and deaths from firearms are uncommon compared to the rest of the world. Additionally, all healthcare facilities should confirm their response plans to emergencies and violence.

Thirdly, the psychological impact of this case on the public is also immeasurable. As cases involving firearms in Japan are few, the public is now being exposed to information containing violence with unprecedented realism. Additionally, cases of COVID-19 in Japan are increasing, potentially placing an additional psychological burden on the general public and healthcare workers, including medical students ^(4)^.

Hence, it is not an overstatement that Japan and the world are facing a public health crisis. Yet, Japan has recovered strongly against disasters and has endured them more resiliently as a society ^(5)^. It is hoped that the world will recover from this abhorrent incident even more strongly in the future.

## Article Information

### Conflicts of Interest

None

### Author Contributions

The author is responsible for the conceptualization and the writing of the manuscript.

### Approval by Institutional Review Board (IRB)

Not applicable
